# Contracture Knots vs. Trigger Points. Comment on Ball et al. Ultrasound Confirmation of the Multiple Loci Hypothesis of the Myofascial Trigger Point and the Diagnostic Importance of Specificity in the Elicitation of the Local Twitch Response. *Diagnostics* 2022, *12*, 321

**DOI:** 10.3390/diagnostics12102365

**Published:** 2022-09-29

**Authors:** Jan Dommerholt, Robert D. Gerwin

**Affiliations:** 1 Bethesda Physiocare, Bethesda, MD 20814, USA; 2 Myopain Seminars, Bethesda, MD 20814, USA; 3 Department of Physical Therapy and Rehabilitation Science, School of Medicine, University of Maryland, Baltimore, MD 21201, USA; 4 Department of Neurology, Johns Hopkins University, Baltimore, MD 21205, USA

**Keywords:** trigger point, contracture knot

## Abstract

A recent study published in *Diagnostics* attempted to visualize trigger points and contracture knots with high-definition ultrasound. Based on their findings, the authors reversed the commonly understood meaning of the two terms. However, they did so without providing any convincing evidence. The authors maintained that their sonography images represented trigger points within contracture knots, supporting the multiple loci hypothesis. On review of the paper, both conclusions seem premature and rather speculative.

## 1. Introduction

In a recent study published in *Diagnostics*, Ball et al. [1] attempted to visualize trigger points and contracture knots (foci of segmental sarcomere contraction [2]) using high-definition sonography. While potentially the study could contribute to a better understanding of myofascial trigger points (TrPs), the paper’s conclusions are not supported by the data provided by the authors. Furthermore, the authors claimed that a “lack of clarity in objective definition” of TrPs and contracture knots had resulted in “unnecessary communication difficulties between and among clinicians and researchers” [1], but they did not include any indication when and where this confusion might have occurred. They may have contributed further to the perceived lack of clarity by not only contradicting themselves, but also by inadvertently redefining the two terms.

For the record, prior to preparing this comment, the first author did communicate with the first author of the paper in question to clarify their intentions. We have collaborated previously with the authors in several other publications [3,4,5,6,7,8,9] and continue to collaborate.

## 2. Background

There is a general consensus in the myofascial pain literature that “a TrP is a hyperirritable spot in a taut band of a skeletal muscle that is painful on compression, stretch, overload or contraction of the tissue, which usually responds with a referred pain that is perceived distant from the spot [10]. An international Delphi panel proposed that at least two of the following criteria must be present for TrP diagnosis: a taut band, a hypersensitive spot, and referred pain [9]. From an etiologic perspective, a TrP can be described as “a cluster of electrically active loci each of which is associated with a contraction knot and a dysfunctional motor endplate in skeletal muscle” [11]. In other words, a palpable TrP is considered to consist of multiple contractions or preferably, contracture knots, which are not palpable, but conceivably can be targeted with dry needling or TrP injections [12]. The term *contraction knot* is more commonly used than the term *contracture knot*, but since taut bands may be contractures and not contractions, contracture knot may be a more accurate term. The nature of the taut band, however, has not been definitively determined, and there may be alternative explanations to explain the finding of a taut band.

Contracture knots are considered to be associated with dysfunctional motor endplates featuring an excessive release of acetylcholine, decreased acetylcholinesterase activity, and increased responsiveness of acetylcholine receptors [13]. The best data to support this contention comes from animal studies, though they may not fully resemble the human situation. In a rodent model, dry needling restored the function of motor endplates, normalizing the release of acetylcholine, reducing the sensitivity of cholinergic receptors, restoring the function of acetylcholinesterase, and significantly decreasing the amplitudes and frequencies of endplate noise and endplate spikes observed in TrPs [14].

## 3. Results

### 3.1. Hyperperfusion

Ball et al. reported that researchers at the US National Institutes of Health identified “ischemia and hypoxia within TrP areas with surrounding hyperperfusion”, which is an inaccurate citation and interpretation of the referenced studies [15,16]. While an area of hyperperfusion in the immediate vicinity of TrPs appeared as hypoechoic on ultrasound, Sikdar et al. did not examine the degree of perfusion *within* TrPs [15], unlike Brückle et al., who, in 1990, observed hyperperfusion outside TrPs and hypoperfusion within TrPs [17].

The second paper cited by Ball et al. applied a microanalytic technique with immunocapillary electrophoresis and capillary electrochromatography to investigate the biochemical milieu of the upper trapezius muscle in subjects with active, latent, or absent TrPs compared to the gastrocnemius muscle, and did not include any perfusion studies [16]. Yet, Ball et al. contrasted Sikdar et al.’s findings with “previous US studies” that “had instead identified and labeled TrPs as hypoechoic”. The two cited papers were neither published previously, nor contradicted Sikdar et al.’s 2010 publication [18]. Mazza et al.’s systematic review was published in 2021, and accurately quoted Sikdar et al.’s findings [18] as did Duarte et al. (2021), who never mentioned hypoechoic areas other than when referencing Sikdar et al. [19].

### 3.2. Interchangeable Use of the Terms “Contracture Knots” and “Trigger Points”

Next, Ball et al. maintained that “the terms “contracture knot” and “TrP” have been used interchangeably,” which in their view constituted a “lack of clarity in objective definition of these two terms,” leading “to unnecessary communication difficulties among clinicians and researchers.” Ball et al. referenced a historical review of the field by Shah et al., which mentioned that “a trigger point complex in a taut band of muscle is composed of multiple contraction knots” [20]. A second reference cited by Ball et al. aimed to “investigate the histopathology of the MTrPs under a transmission electron microscope (TEM) and an optical microscope” [21], which implies they started with the clinical palpable TrP and examined its histopathology. They determined that “the number of contracture knots (CKs) and muscle cell diameters were quantified in a transverse section of each slide under light microscopy using a computer system” [21].

Ball et al. cited two older papers by Hong [22] and Hong and Simons [23], in support of the notion that “sensorimotor abnormalities of TrPs are related to multiple sensitized afferent nerves and motor endplates contained within the “TrP region”, or what clinicians define as a palpable “contracture knot”. In this use of the term, clinicians may be referring to a small focus of hardness within a taut band, which is something different from a non-palpable contracture knot that Ball et al. are referring to in their study. Indeed, in a review article, Hong and Simons maintained that a TrP region would maintain multiple minute sensory loci, possibly sensory receptors or sensory nerve fibers, and active loci, but they did not suggest that these would be palpable and referred to as contracture knots by clinicians [23].

### 3.3. TrP Speckles

Ball et al. included ultrasound images of “TrP speckles” within contracture knots, based on the apparent assumption that contracture knots would present as larger hypoechoic (hyperperfused) areas and TrPs as small hyperechoic (hypoperfused) spots [1]. They maintained to have visualized TrP speckles within contracture knots [1], and postulated that a hypoechoic/hyperperfused area of approximately 1 cm × 1 cm in the vicinity of the palpable nodule represented a contracture with smaller hyperechoic/hypoperfused and nonpalpable TrP speckles of approximately 1 mm × 1 mm within each [1].

## 4. Discussion

It appears that Ball et al. misinterpreted the studies by Sikdar et al., who did not examine the degree of perfusion *within* TrPs [15], which, as we speculate, may have contributed to their thoughts about hyperperfusion, contracture knots, and TrPs. In our humble opinion, Ball et al. did not provide any evidence of an alleged interchangeable use of the terms ‘contracture knot’ and ‘TrPs’ and the communication difficulties this may have caused. Ironically, the authors used the terms interchangeably themselves [1], e.g., when they referenced a 1999 diagnostic ultrasound study for detecting active TrPs [24]. Ball et al. noted that the study failed to identify contracture knots, but the objective of the study was “to find an objective and reliable tool that could identify TrPs” without ever referring to contracture knots [24]. Furthermore, Ball et al. stated that “recent studies on animal models support the idea that contracture knots are collections of smaller TrPs located at the neuromuscular junctions,” [1] citing Liu et al. [13]. While Liu et al. confirmed the presence of contraction knots at motor endplates in an animal model that included blunt trauma to induce the muscle findings, they did not suggest that these contraction knots should be referred to as TrPs [13].

Even in the case reports provided by Ball et al., the authors reversed the commonly used terms when they stated that “we visualized that the palpable and hyperperfused contracture knot is actually a collection of much smaller nonpalpable hypoperfused TrPs at motor endplates residing within the palpable contracture knot” [1]. The authors stated that they identified taut bands with palpable nodules within the medial gastrocnemius, the right anterior and middle deltoid, and the left upper trapezius muscles [1]. In all these cases, they subsequently referred to these palpable nodules as hypoechoic contracture knots. However, in the more common TrP terminology, these would have been described as hypoechoic TrPs.

In the discussion section, Ball et al. confirmed their erroneous assumption that the common terminology used in the myofascial pain literature is incorrect and reversed the terms TrP and contracture knot, stating that “previous studies may have mislabeled contracture knots as TrPs” [1]. The authors suggested “that the relatively large hypoechoic structures visualized as the TrP area in both subjects examined represent hyperperfused contracture knots with smaller, not previously distinguished ischemic and hyperechoic TrPs within each” [1].

On the contrary, we continue to maintain that a TrP is a palpable clinical entity of muscle, which may consist of multiple contracture knots, although we recognize that even a region with multiple contracture knots may not be a palpable entity comparable to what clinicians may call a contraction knot. Palpation of a TrP does not assume any knowledge of presumed underlying pathology; it is comparable to identifying an enlarged liver, which offers no details of its underlying pathology either. Biochemical analyses have revealed elevated levels of a variety of inflammatory mediators, neuropeptides, catecholamines, and cytokines in the vicinity of TrPs [16,25,26], and it is conceivable that TrPs are at least partially palpable because of neurogenic edema, consistent with the hypoechoic ultrasound finding and with biochemical data.

With regard to the observed speckles, Ball et al. assumed these to be hyperechoic TrPs. However, apparently, they did not consider that the observed “speckles” could possibly represent common fibrous densifications associated with perimysial fascia and collagen, which may have no relation to TrPs [27]. Fibrous tissues are also echogenic as their structure is distinctly different from the cytoarchitecture of muscles [28] and they increase the echo intensity of muscles [29]. Also, the authors offered no explanation as to how they differentiated the observed speckles attributed to TrPs from multiple similar speckles elsewhere in the same images.

We agree with Shah and Gilliams that “the pathophysiology is only beginning to be understood due to its enormous complexity” [30] and a human case series may be premature to truly advance the understanding of the pathophysiology of TrPs. Instead, we recommend using an established animal TrP model [14,21], and biopsy palpable taut bands and TrP zones with serial sections for regions of segmental contractures as suggested by Gerwin et al. [2].

## 5. Conclusions

While we welcome innovative studies of the nature of TrPs, we do not recognize that Ball et al. are providing any evidence of “palpable contracture knots with smaller TrPs within.” Based on personal communication (10 May 2022), Ball et al., apparently aimed to work toward clarification and standardization, and redefining the definitions of contracture knots and TrPs may not have been the intended outcome. Nevertheless, labeling hyperechoic speckles on muscle images as TrPs seems premature and rather speculative.
